# New Porcine Model of Arteriovenous Fistula Documents Increased Coronary Blood Flow at the Cost of Brain Perfusion

**DOI:** 10.3389/fphys.2022.881658

**Published:** 2022-04-27

**Authors:** Anna Valerianova, Mikulas Mlcek, Tomas Grus, Jan Malik, Otomar Kittnar

**Affiliations:** ^1^ 3rd Department of Internal Medicine, General University Hospital in Prague, 1st Faculty of Medicine, Charles University, Prague, Czechia; ^2^ Institute of Physiology, 1st Faculty of Medicine, Charles University, Prague, Czechia; ^3^ 2nd Surgical clinic, Cardiovascular Surgery, General University Hospital in Prague, 1st Faculty of Medicine, Charles University, Prague, Czechia

**Keywords:** arteriovenous fistula, hyperkinetic circulation, tissue perfusion, animal model, cerebral oxygenation, coronary artery flow

## Abstract

**Background:** Arteriovenous fistulas (AVF) represent a low resistant circuit. It is known that their opening leads to decreased systemic vascular resistance, increased cardiac output and other hemodynamic changes. Possible competition of AVF and perfusion of other organs has been observed before, however the specific impact of AVF has not been elucidated yet. Previous animal models studied long-term changes associated with a surgically created high flow AVF. The aim of this study was to create a simple AVF model for the analysis of acute hemodynamic changes.

**Methods:** Domestic female pigs weighing 62.6 ± 5.2 kg were used. All the experiments were held under general anesthesia. The AVF was created using high-diameter ECMO cannulas inserted into femoral artery and vein. Continuous hemodynamic monitoring was performed throughout the protocol. Near-infrared spectroscopy sensors, flow probes and flow wires were inserted to study brain and heart perfusion.

**Results:** AVF blood flow was 2.1 ± 0.5 L/min, which represented around 23% of cardiac output. We observed increase in cardiac output (from 7.02 ± 2.35 L/min to 9.19 ± 2.99 L/min, *p* = 0.0001) driven dominantly by increased heart rate, increased pulmonary artery pressure, and associated right ventricular work. Coronary artery flow velocity rose. On the contrary, carotid artery flow and brain and muscle tissue oxygenation measured by NIRS decreased significantly.

**Conclusions:** Our new non-surgical AVF model is reproducible and demonstrated an acute decrease of brain and muscle perfusion.

## Introduction

An arteriovenous fistula (AVF) represents a low-resistant circuit bypassing the resistant arterioles (Malik, Tuka, and Tesar 2009). AVFs could be congenital, post-traumatic or created intentionally as a vascular access for hemodialysis (Fila et al., 2020). Creation of a large arteriovenous fistula leads to a sudden decrease in systemic vascular resistance, increase in venous return, increase in cardiac output and other hemodynamic changes, that have been described previously by Guyton and others (Guyton and Sagawa 1961).

High-flow peripheral AVF or aortocaval fistula could lead to heart failure (HF) development, which is used in animal HF models especially in rats (Melenovsky et al., 2011), but also in dogs (Habibullah et al., 1995) or pigs (Modesti et al., 2004). Although rats are easily accessible and create less ethical issues, their physiology differs from larger animals and human beings. One such example is the reaction to volume overload: in rats, the increase in cardiac output (CO) is accomplished solely by higher stroke volume and not by increased myocardial contractility that is known for humans and pigs at least during weeks and months (Liu, Hilbelink, and Gerdes 1991). This fact together with our previous experience with pig biomodels (Ostadal et al., 2018; Hala et al., 2020; Popkova et al., 2020), led to the selection of the swine model.

The arterial tree in mammals includes both high- and low-resistant beds. High peripheral arterial resistance is typical for the extremities at rest. Low-resistant circuit ensures relatively stable blood supply into vital organs such as brain, kidneys, and coronary arteries. Since AVF is also a low-resistant circuit, its competition with other low-resistant beds is feared. Indeed, first human data suggested negative effect of an AVF on transplanted kidney perfusion (Laranjinha et al., 2019) and on brain oximetry (Kovarova et al., 2021), improving during manual AVF compression. In patients after left mammary artery-left anterior descending artery bypass, ipsilateral upper extremity AVF can cause worsening of angina pectoris (Feldman et al., 2014). However, the effects of AVF creation on brain or myocardial perfusion remains to be elucidated.

Infrarenal aortocaval fistula creation is also possible in porcine models, but surgical approach to the retroperitoneum is needed (Modesti et al., 2004). We therefore looked for another option–a more peripheral AVF that would be easier to perform and that would increase the cardiac output sufficiently and offer reproducible results. AVF flow volume depends (besides the mean arterial pressure) on peripheral vascular resistance where the size of the arteriovenous anastomosis plays the most important role (Sullivan et al., 1993). We presumed that prefabricated tube with a constant orifice area could be used for such AVF. Our lab has considerable experience with the use of the extracorporeal membrane oxygenation (ECMO) in pigs (Ostadal et al., 2018; Hala et al., 2020; Popkova et al., 2020), thus we have tested several ECMO cannulas and sets for creating an AVF.

The aims of this study were as follows: 1. To create a non-surgical model of AVF in pigs giving sufficient and reproducible AVF flow volume; 2. To describe acute changes in organ and tissue perfusion in response to AVF creation in stable state.

## Materials and Methods

The study was performed in The Common Experimental Laboratory of the Department of Physiology, First Faculty of Medicine, Charles University, Prague, on female domestic pigs (*Sus scrofa domestica*). The animals were handled in accordance with the guidelines and legal requirements for animal use in research. The study was approved by Institutional care and use committee and was performed in accordance with European Guidelines on Laboratory Animal Care.

### The Protocol

11 healthy swine were included into the study, weighing 62.6 ± 5.2 kg. All the experiments were held under general anesthesia. After premedication with intramuscular (i.m.) injection of midazolam (0.3 mg/kg) and ketamine (20 mg/kg), the marginal ear vein was cannulated. After preoxygenation with 100% oxygen *via* a facial mask, the general anesthesia was induced with intravenous bolus (i.v.) of propofol (1–2 mg/kg) followed by a continuous infusion. The orotracheal intubation was performed and mechanical ventilation started. During the procedure, the ventilation was adjusted to maintain normoxia (peripheral oxygen saturation ≥97%, pO_2_ 100 mmHg) and normocapnia (EtCO_2_ 38–40 mmHg). During the whole experiment, total intravenous anesthesia was maintained by a continuous intravenous infusion of propofol, morphine and midazolam with regular controls of pupillary and corneal reflexes to monitor the depth of anesthesia. The anesthesia was adjusted before starting the protocol itself and then remained unchanged to avoid the effect of dose variation of anesthetics on cardiovascular system. Anticoagulation was maintained by unfractionated heparin, starting with i. v. bolus 100 units per kilogram followed by continuous i. v. infusion to reach the target activated clotting time (200–250 s). No inotropes or vasopressors were used during the experiment.

Sheaths and catheters were inserted into jugular and femoral veins and femoral artery for later placement of measurement catheters.

**FIGURE 1:**
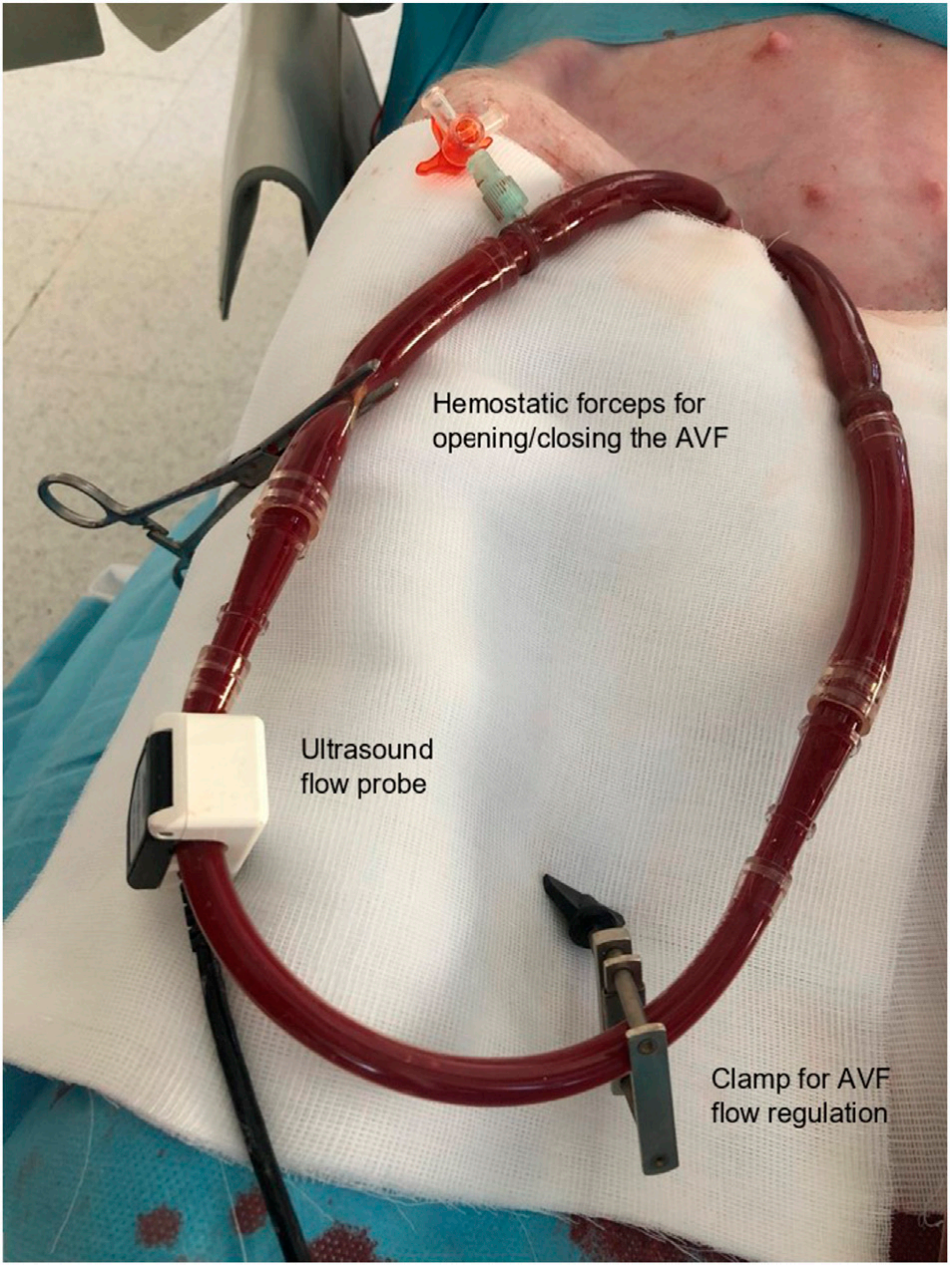
Aortocaval arteriovenous fistula created by the connection of two high-diameter ECMO cannulas. Special clamp to regulate the AVF blood flow volume was placed before connecting the ECMO cannulas (in lower right corner). Continuous measurement of AVF flow was performed using an ultrasound probe (arrow) (Transonic, United States). This photo documents clamping of the fistula circuit by the hemostatic forceps for the observation of the hemodynamic stabilization in the final phase of the experiment.

AVF was created by the connection of femoral artery and femoral vein using percutaneously inserted high-diameter perfusion cannulas (18 F for the arterial cannula and 23 F for venous cannula), see Figure 1. AVF blood flow was continuously measured by transient-time ultrasound probe fixed to the ECMO set (Transonic, United States). A special clamp fixed around the arterial cannula allowed us to regulate the AVF blood flow and to close the fistula when required, see Figure 1. Initial AVF flow measurement was performed to verify a sufficient AVF functioning.

**FIGURE 2:**
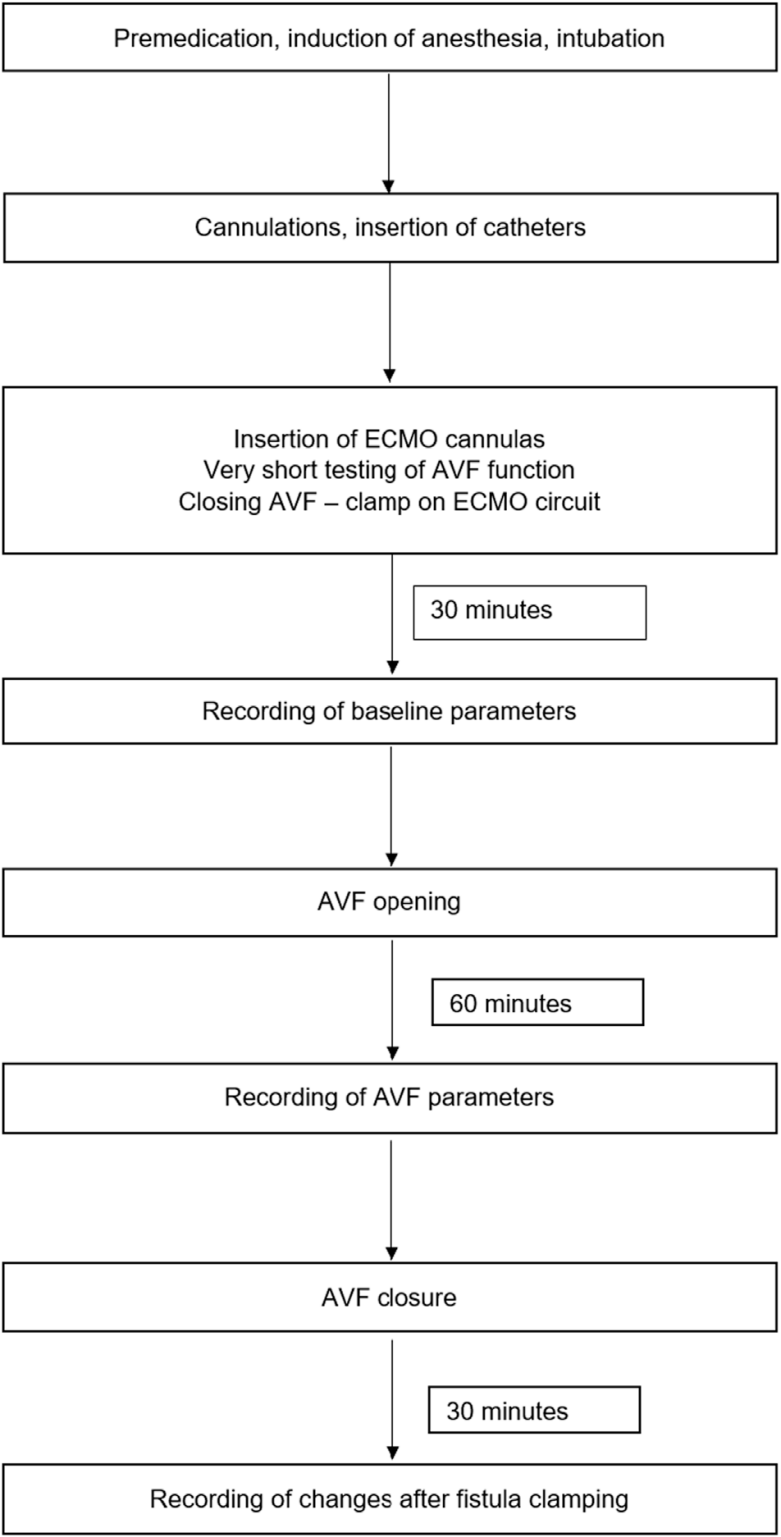
Scheme of study protocol.

Afterwards, the AVF was closed by the clamp and after 30 min of stabilization the baseline values of were recorded. The fistula was then reopened and AVF data were collected after animal stabilization after at least 1 hour of observation. The timeframe of the study is displayed in Figure 2.

### Measured Parameters

Continuous invasive arterial blood pressure measurement was performed using a monitoring catheter inserted in contralateral femoral or radial artery. Central venous pressure was measured using the central venous catheter inserted in the left jugular artery. Pulmonary arterial pressure, continuous measurement of mixed venous oximetry (SvO_2_) and cardiac output measurement based on thermodilution method were measured and recorded using the Swan-Ganz catheter inserted in pulmonary artery under X-ray control *via* the right jugular vein. Stroke volume was calculated as *SV = CO/HR* (SV–stroke volume, CO–cardiac output, HR–heart rate). Continuous surface ECG, peripheral oxygen saturation and EtCO_2_ monitoring was performed. Left ventricular stroke work was approximated as *SVx*(*MAP-PCW*)*x0.0136* (SV–stroke volume, MAP–mean arterial pressure, PCW–pulmonary capillary wedge pressure). Analogously, right ventricular work was calculated as *SVx*(*PAMP-CVP*)*x0.0136* (PAMP–pulmonary artery mean pressure, CVP–central venous pressure). Carotid artery flow was continuously measured by transient-time perivascular ultrasound probe (Transonic, United States). The intracoronary artery flow was measured by a Doppler flow wire (FloWire, Volcano, United States) inserted into the left anterior descending or circumflex artery approximately 8 cm distal to left coronary artery ostium. Measurement of regional tissue oxygen saturation (rSO_2_) was performed using the INVOS 5100C oximetry system (Medtronic), working on the near-infrared spectroscopy (NIRS) principle. Four sensors were placed on the front leg, on the head in the frontal region, on the back and on the hind leg with the arteriovenous fistula.

### Data Collection and Statistics

Most of the data were real-time recorded and stored on a PC using ADI PowerLab ADC and LabChart Pro software (ADInstruments, New Zealand) with sampling rate 10 kHz. INVOS data were recorded every 6 s. The data were analyzed using the Statistica software (StatSoft Inc., United States). Paired t-test or ANOVA were used as appropriate. Correlation analysis was performed according to Pearson. Continuous variables are presented as mean ± SD. The *p*-value < 0.05 was considered significant.

## Results

**FIGURE 3:**
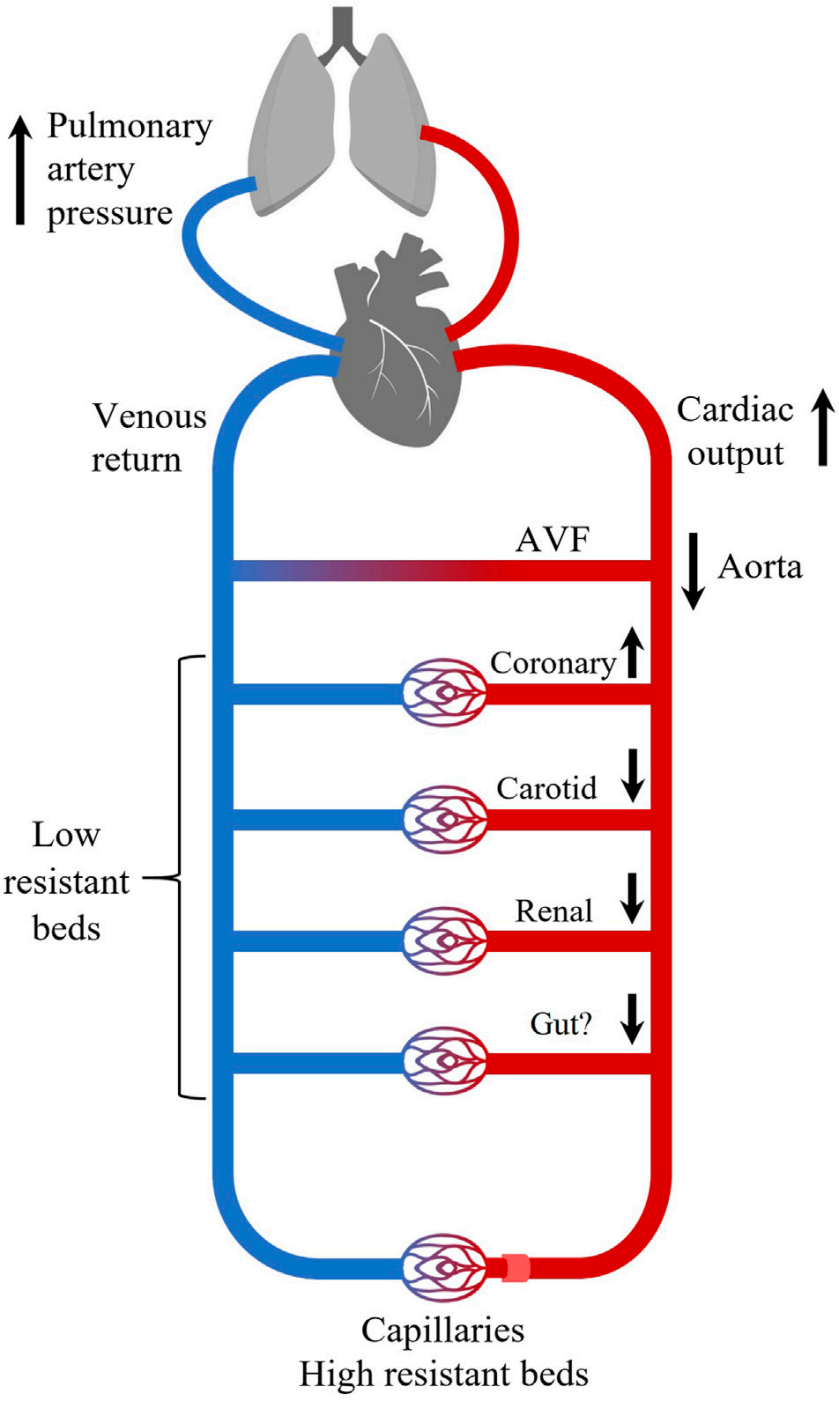
The acute effects of high-flow AVF on systemic hemodynamics. Opening of a high-flow arteriovenous fistula increases cardiac output (CO) and coronary blood flow at cost of perfusion of other vascular beds (brain, kidneys, peripheral tissues). We did not measure gut perfusion, but it could be influenced too. Increase in CO leads to increase in pulmonary artery pressure. High resistant beds refer to arteries supplying extremities and other striated muscles. Arrows indicate increase/decrease of blood flow.

11 healthy swine were included into the study. The AVF blood flow volume (Qa) was 2089 ± 513 ml/min. It represented 23 ± 4% of baseline cardiac output. Effects of AVF opening are summarized in Figure 3.

### Hemodynamics

**FIGURE 4:**
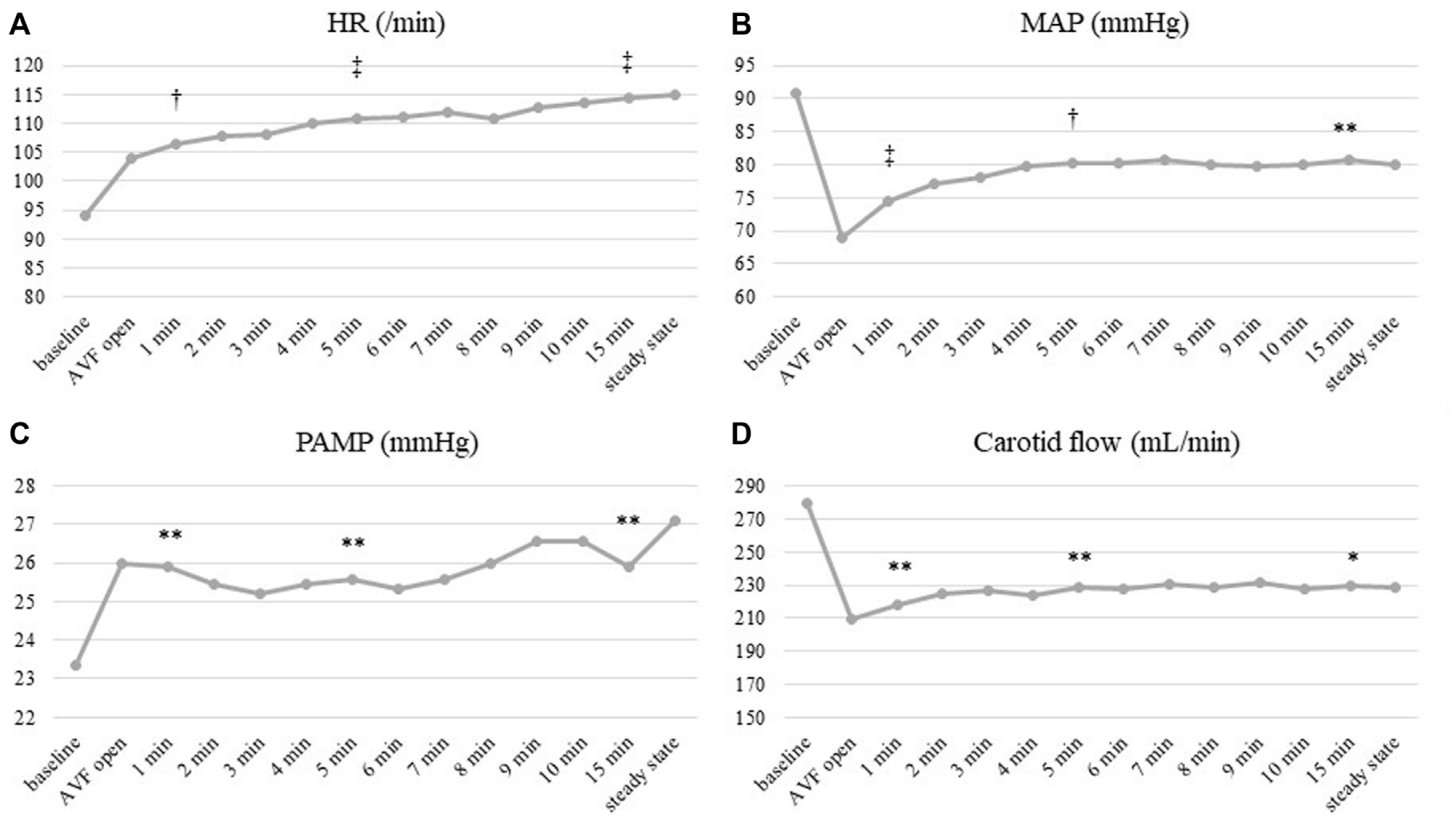
Time course of hemodynamic parameters after AVF opening. **(A)** Heart rate, **(B)** Mean arterial pressure (MAP), **(C)** Pulmonary artery mean pressure (PAMP), **(D)** Carotid artery flow. HR and PAMP **(A,C)** increased immediately after AVF opening and showed a continuous increase afterwards. MAP and carotid artery flow **(B,D)** dropped rapidly, then rose after the initial dip; the resulting values remained lower than in baseline. Significance of observed changes after AVF opening *vs*. baselines was calculated at 1, 5 and 15 min after AVF opening. Displayed values are averaged from all subjects. *p < 0.05; **p < 0.01; †*p* < 0.001; ‡*p* < 0.0001.

Hemodynamic changes following the creation of AVF after 60 min of model stabilization are displayed in Table 1. Changes of main hemodynamic parameters immediately after AVF opening are displayed in Figure 4. Heart rate and pulmonary artery pressure showed a continuous increase since AVF opening (Figures 4A,C). Mean arterial pressure dropped rapidly, then rose after the initial dip; however, the resulting values remained lower than in baseline (Figure 4B).

**TABLE 1:** Hemodynamic changes after AVF creation.

Parameter	Baseline	AVF	*p*-value
Qa (L/min)	—	2.1 ± 0.5	—
HR (/min)	94 ± 21	111 ± 25	**0.0005**
ART(S) (mmHg)	117 ± 15	107 ± 21	**0.03**
ART(D) (mmHg)	77 ± 13	63 ± 15	**0.002**
ART(M) (mmHg)	89 ± 13	77 ± 16	**0.0007**
CVP (mmHg)	4 ± 2	4 ± 2	0.11
PASP (mmHg)	32 ± 10	37 ± 8	**0.04**
PAMP (mmHg)	23 ± 7	29 ± 9	**0.02**
PCWP (mmHg)	9 ± 4	7 ± 4	0.32
SvO_2_ (%)	59.6 ± 11.6	66.1 ± 9.9	**0.006**
CO (L/min)	7.02 ± 2.35	9.19 ± 2.99	**0.0001**
% of CO through AVF	—	23 ± 4	—
SV (ml)	76 ± 20	84 ± 21	0.08
SVR (WU)	13.3 ± 4.3	11.1 ± 3.4	**0.018**
PVR (WU)	3.6 ± 1.4	3.3 ± 1.0	0.26
LV work (gm)	86.3 ± 30.9	82.1 ± 32.8	0.45
RV work (gm)	19.9 ± 6.5	27.2 ± 8.8	**0.007**
Carotid flow (ml/min)	264 ± 137	215 ± 105	**0.002**
Coronary flow velocity (cm/s)	21.1 ± 12.2	25.5 ± 12.3	**0.000007**

Statistically significant results are in bold. Displayed values are averaged from all subjects. “AVF” data represent measurements obtained after 60 min of opened AVF after reaching stable state. Qa, arteriovenous fistula blood flow; HR, heart rate; ART, systemic arterial blood pressure; S, systolic; D, diastolic; M, mean; CVP, central venous pressure; PASP, pulmonary artery systolic pressure; PAMP, pulmonary artery mean pressure; PCWP, pulmonary capillary wedge pressure; SvO_2_, hemoglobin saturation in mixed venous blood; CO, cardiac output; SV, stroke volume; SVR, systemic vascular resistance; PVR, pulmonary vascular resistance; WU, Wood units; LV, left ventricle; RV, ight ventricle.

### Heart

Mean coronary artery blood flow velocity increased from 21.1 ± 12.2 cm/s to 25.5 ± 12.3 cm/s (*p* = 0.000007). The increase was directly proportional to increase in cardiac output (R = 0.66; *p* = 0.038). We observed associated rise in right ventricular work that was related to increased mean pulmonary artery pressure (R = 0.91, *p* = 0.000). There was no significant correlation of RV work with stroke volume (R = -0.085, *p* = 0.80).

### Brain

Carotid artery flow decrease showed a similar pattern to mean arterial pressure–fast initial drop after AVF opening, following by slow increase, but staying lower than in baseline (264 ± 137 ml/min *vs*. 215 ± 105 ml/min; *p* = 0.002) (Figure 4D). Cerebral rSO_2_ decreased significantly with AVF opened (from 63.5 ± 7.8% to 60.4 ± 9.5%; *p* = 0.037; see Table 2). Both carotid artery flow and cerebral rSO_2_ increased after AVF closure, returning back to baseline values (carotid blood flow to 267 ± 141 ml/min, cerebral rSO_2_ to 63.5 ± 8.1%).

**TABLE 2:** Changes in tissue perfusion and oxygenation after AVF opening.

Parameter	Baseline	AVF	*p*-value
NIRS (%)			
Head	63.5 ± 7.8	60.4 ± 9.5	**0.037**
Front leg	56.9 ± 5.1	54.3 ± 5.4	**0.019**
Back	51.7 ± 7.9	49.2 ± 6.4	**0.049**
Hind leg	48.7 ± 6.7	45.7 ± 11.4	0.13

Statistically significant results are in bold. Displayed values are averaged from all subjects. “AVF” data represent measurements obtained after 60 min of opened AVF after reaching stable state. NIRS, near infrared spectroscopy.

### Other Organs and Tissues

We observed a significant decrease of tissue oxygenation measured by NIRS of the front leg and of the body after AVF opening. No change was observed for the hind leg with introduced ECMO cannulas; see Table 2. However, the diameter of the ECMO cannula was almost equal to the diameter of the femoral artery (6–7 mm), thus the values were possibly influenced by obturation of the femoral artery.

**FIGURE 5:**
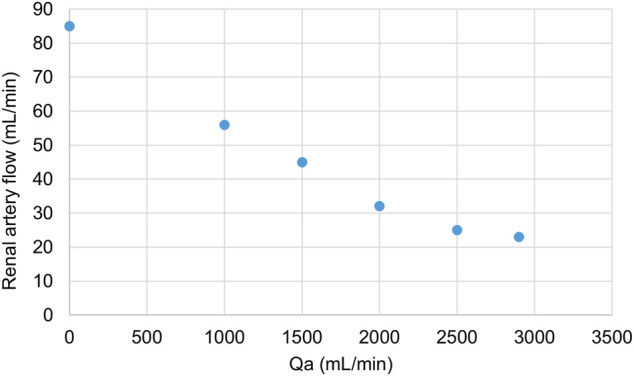
Changes in renal artery flow with increasing Qa. Displayed values were obtained from one subject. Qa was regulated by a clamp on the ECMO set.

In one of our animals, laparotomy was performed, and ultrasound flow probe was placed around right renal artery. The blood flow gradually decreased with increasing of the AVF flow, see Figure 5.

## Discussion

We have created a reproducible porcine model of aortocaval fistula using percutaneous arterial and venous access, thus avoiding the need of a surgical approach. Using this new model, we documented that the increase in CO was linked with higher coronary blood flow, but at the cost of lower carotid perfusion and brain oxygenation.

Animal models of aortocaval arteriovenous fistulas are well-known for past decades. Large AVFs are used not only to study hemodynamics of hyperkinetic circulation, but also as an easily obtainable model of HF. They are usually created by a direct connection of aorta an inferior vena cava (Modesti et al., 2000; Duggan and Tabrizchi 2016). However, all these techniques require participation of a skilled (vascular) surgeon. Moreover, the intra-abdominal location of the fistula also makes measurement of blood flow technically challenging. Indeed, most studies did not present AVF flow (Taylor, Covell, and Ross 1968; Modesti et al., 2000). The use of ECMO cannulas brought the advantage of a constant size of the arterial anastomosis and their introduction is less invasive. The disadvantage of our model is that it can be used only in non-surviving experiments under general anesthesia and thus it could be only used for studying acute AVF effects.

Cardiac output increases immediately after AVF opening according to Guyton (Guyton and Sagawa 1961). The rise in cardiac output in our study was driven mainly by higher heart rate; the increase in stroke volume was not significant in contrast to other studies (Modesti et al., 2004). This documents rather the effect of acute sympathetic activation, but we have to admit that the stroke volume was measured only indirectly in our study. Bainbridge reflex (heart rate increase due to higher atrial filling) is an alternative explanation; however, we did not observe a change in atrial pressure. We hypothesize that the stroke volume increase develops later as other studies used animal surviving experiments (Modesti et al., 2004).

We observed an increase in coronary blood flow velocity after opening of AVF that related to the rise in cardiac output. Coronary blood flow is known to be determined dominantly by metabolic demands of the cardiac muscle. Coronary oxygen extraction is high even at rest and reaches up to 70% (Duncker and Bache 2008). Therefore, every increase in oxygen consumption in the myocardium leads to increase in coronary blood flow (Duncker et al., 2015). Oxygen consumption and coronary blood flow increase dominantly with rise in heart rate and mechanical work of the heart (Graham et al., 1968; McGinn, White, and Wilson 1990)—as in this study. Hence the increase in coronary blood flow velocity can be explained by a physiological compensation of higher myocardial metabolism. Our study used Doppler wire for the detection of coronary artery flow velocity, not directly coronary blood flow. However, rising blood velocity increases the wall shear stress (Gnasso et al., 2001), so we can presume that the coronary diameter (another determinant of flow) rose as well - or at least remained the same.

Carotid arterial flow and cerebral tissue oxygen saturation (rSO_2_) decreased after AVF opening in our study. As we are aware that propofol could affect cerebral blood flow (Lagerkranser, Stange, and Sollevi 1997), we did not change the dose of anesthesia during the protocol and took the control values of carotid flow velocity and cerebral rSO_2_ 30 min after AVF closure. Their return to baseline values allows us to say that propofol did not play any role in the observed changes. Although human and pig brains differ considerably, these observations are in accord with our human data: hemodialysis patients showed lower cerebral rSO_2_ than in healthy controls (Malik et al., 2017) with a significant increase after short-term AVF compression (Kovarova et al., 2021). Flow reduction surgery in high-flow arteriovenous fistulas led to increased cerebral rSO_2_ in humans in our recently published study (Malik et al., 2021). Still, the effects of AVF creation and flow on the brain have to be elucidated: our study did not measure neither the level of brain oxygen extraction, nor invasive tissue flow or oxygenation changes and observed only acute changes.

Another example of competition of low resistant vascular beds is the decrease of renal perfusion. Although we have data available only from one animal, there was a clearly visible decrease of renal artery blood flow while increasing the Qa. As mentioned before, temporary occlusion of AVF led to higher perfusion of graft in kidney transplant recipients (Laranjinha et al., 2019). The fall of systemic blood flow after AVF opening was documented before (Guyton and Sagawa 1961). Our results–specifically carotid artery flow decrease–suggest that competition is present even in the brain and kidneys, but more detailed data are needed to support this hypothesis.

Both ventricles are exposed to volume overload when an AVF is created. However, the right ventricle also faces the increased pressure load, caused by increased pulmonary arterial pressure. Both these factors led to a significant increase in right ventricular work. Indeed, right ventricular free wall thickening was documented in a study performed at fetal sheep (Karamlou et al., 2019). Cardiac hypertrophy is a well know consequence of AVF creation, but most studies are oriented at the size and weight of the whole heart or on the left ventricle.

Possible clinical implications of our study are as follows: 1. Decreased systemic steal of non-coronary arterial beds could contribute to their known worsened function, for example, in case of brain in patients on chronic hemodialysis (Michna et al., 2020); 2. Cardiac output distribution could be even more different in critical states or during cardio-surgical procedures “on pump”.

Vast majority of data describing hemodynamics of high-flow arteriovenous fistulas were conducted on patients or animal models in stable state. The impact of AVF in critical states (e.g., cardiac surgery with extracorporeal circulation, sepsis, shock, effect of catecholamines) is unknown. We assume, that a low-resistance arteriovenous shunt can compete with other low-resistance parts of circulation in unstable conditions. To be able to study these critical states in AVF model, we needed to create a suitable animal model. Nothing is known about the changes during physical exercise that is associated with decreased peripheral vascular resistance of arteries supplying striatd muscles. This could lead to more pronounces competition of low-resistant beds.

Our study has its limitations. Firstly, we performed only a short-term study to describe acute haemodynamic changes. Thus, long-time adaptation mechanisms (e.g., changes in stroke volume or later decrease of peripheral vascular resistance) did not have time to develop. Secondly, as we state earlier, we were not able to invasively measure cerebral tissue flow or partial tissue oxygen pressure. However, same directions of carotid blood flow and of brain oxygenation make this unfavorable effect on brain perfusion more probably.

We succeeded in creating an easily reproducible model of hyperkinetic circulation caused by arteriovenous fistula. The changes in cardiac output, brain perfusion and cerebral tissue oxygenation are in accordance with our observations in patients with high-flow AVFs published previously (Malik et al., 2021; Valerianova et al., 2021). This allows us to state that the model should be suitable for performing subsequent studies focused on critical conditions.

## Data Availability

The raw data supporting the conclusions of this article will be made available by the authors, without undue reservation.

## Abbreviations

AVF, arteriovenous fistula; CO, cardiac output; CVP, central venous pressure; ECG, electrocardiogram; ECMO, extracorporeal membrane oxygenation; EtCO_2_, end-tidal carbon dioxide pressure; HF, heart failure; HR, heart rate; i.m., intramuscular; i.v., intravascular; MAP, mean arterial pressure; NIRS, near-infrared spectroscopy; PAMP, pulmonary artery mean pressure; PCWP, pulmonary capillary wedge pressure; pO_2_, partial pressure of oxygen; Qa, arteriovenous fistula blood flow volume; rSO_2_, regional tissue oxygen saturation; SV, stroke volume; SvO_2_, mixed venous blood oxygen saturation.
